# Basal cell adenoma and basal cell adenocarcinoma of the parotid gland: clinical findings and surgical outcomes in a single-institution study

**DOI:** 10.1186/s12957-024-03378-6

**Published:** 2024-04-18

**Authors:** Chongsoo Park, Sally Min, Joseph Kyuhyung Park, Jong-Ho Kim

**Affiliations:** 1 Department of Plastic and Reconstructive Surgery, Inje University Busan Paik Hospital, Inje University College of Medicine, Busan, Korea; 2 Department of Plastic and Reconstructive Surgery, Seoul National University College of Medicine, Seoul National University Bundang Hospital, 82 Gumi-ro 173beon-gil, Bundang-gu, Seongnam, 463-707 Korea

**Keywords:** Basal cell adenoma, Basal cell adenocarcinoma, Surgery, Tumor

## Abstract

**Background:**

Basal cell adenoma (BCA) is a rare benign tumor within the salivary glands. Basal cell adenocarcinoma (BCAC), the malignant counterpart of BCA, is also an exceedingly rare tumor with very limited clinical studies conducted. This study aims to investigate the clinical characteristics, demographics, and surgical outcomes of patients diagnosed with BCA and BCAC within the parotid gland.

**Methods:**

A retrospective analysis from May 2003 to August 2023 was performed for all patients undergoing parotidectomy for masses. Retrospective data on gender, age, tumor characteristics, and outcomes were collected. Surgical approaches, including negative margin attainment, capsule removal, and histological diagnosis, were also detailed.

**Results:**

The study included 1268 patients who underwent parotidectomy, resulting in 81 cases of BCA and 7 cases of BCAC. BCA patients, with a mean age of 55.1 years, showed diverse age distribution and predominantly presented in the 50s. In BCAC cases, seven female patients exhibited a predominant location in the deep lobes. FNA revealed BCAC in three out of seven cases, and subsequent parotidectomy was performed, resulting in no observed recurrences or metastases.

**Conclusion:**

This study reports the largest number of BCA cases from a single institution and provides comprehensive insights into the demographics, tumor characteristics, and clinical outcomes of both BCA and BCAC. Although further research should be conducted, based on clinical follow-up results, appropriately including the capsule in the tumor excision indicates favorable outcomes, especially when the tumor size is not large.

## Introduction

Basal cell adenoma (BCA) is one of the rare benign tumors occurring in the salivary glands, constituting one of the 11 types of benign tumors in this category [1, 2]. The World Health Organization (WHO) classification categorized BCA as an unspecified entity with an overall incidence rate ranging from 1.1 to 3.7% [3]. In clinical settings, BCA is frequently identified by its characteristics of being a slowly progressing mass, usually asymptomatic, and displaying ease of mobility [4]. Primarily arising in the parotid gland, BCA is distinguished by a unique histological appearance, showcasing monomorphic basaloid cells without a myxochondroid component [5].

Most parotid gland tumors are benign, constituting approximately 90%, while only about 10% are malignant. BCAC, the malignant counterpart of BCA, constitutes 2.9% of all salivary gland carcinomas [6]. Most BCACs were identified in the parotid glands, and due to its rarity, clinical studies and available clinical information are limited [7, 8]. Fine needle aspiration (FNA), a crucial diagnostic tool for assessing parotid masses, offers preliminary insights before surgical intervention—a significance that extends to the diagnosis of BCAC as well [9]. However, BCAC is often reported to pose challenges in differential diagnosis with other parotid tumors, leading to occasional misdiagnosis [10–12].

BCA and BCAC have primarily been documented through case reports and population-based studies [8, 12–16]. Nevertheless, there has been a recent surge in reporting a relatively large number of cases from a single-center-based study [7, 17, 18]. Despite this increase, a notable shortage of studies persists in illustrating the demographic and clinical characteristics of these entities. Therefore, we focused on BCA and BCAC within the parotid gland, aiming to investigate the clinical findings and surgical outcomes in this study.

## Methods

**Fig. 1:**
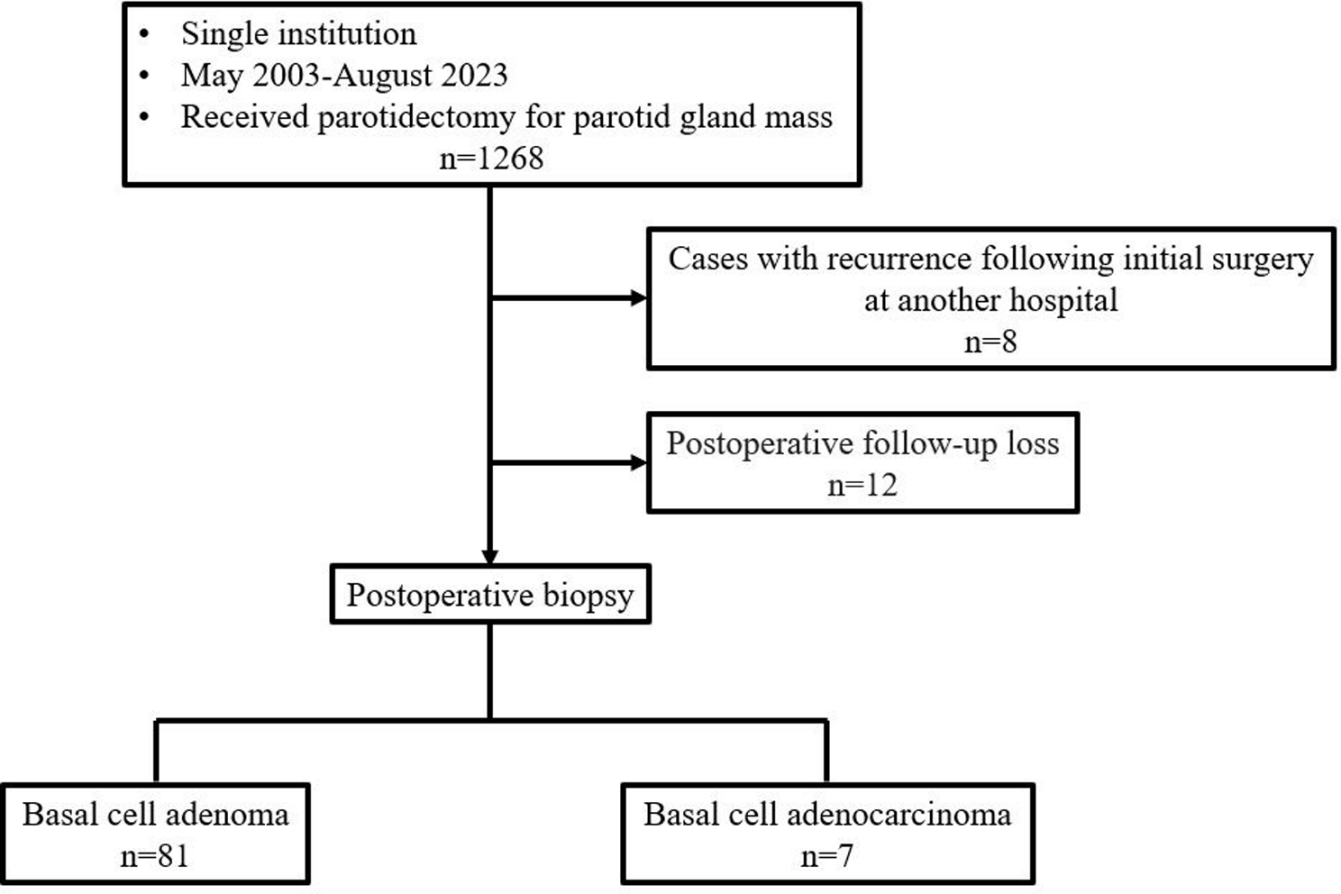
Flow chart of patient selection

We conducted an investigation involving all patients presenting with parotid gland masses and undergoing parotidectomy at our institution from May 2003 to August 2023. This study was approved by the Ethics Committee of Seoul National University Bundang hospital. The exclusion criteria included cases with recurrence following initial surgery at another hospital and instances of postoperative follow-up loss (Fig. 1) Patients with confirmed BCA or BCAC in postoperative biopsies were included in the study. All clinical information, including gender, age, location, size, and follow-up period, was retrospectively collected. Preoperative symptoms, preoperative facial nerve status, type of parotidectomy, histopathological findings, and treatment outcomes including postoperative complications were retrospectively reviewed. In the case of BCAC, the pathologic stage was recorded, and any recurrences or deaths during the follow-up period were also investigated.

### Surgical methods

Patients underwent partial, superficial, or total parotidectomy depending on the tumor’s location. The primary tumor was excised to achieve a negative surgical margin. In BCAC cases, which were confirmed in preoperative FNA, negative margins were confirmed using intraoperative frozen biopsy. Due to the encapsulated nature observed in both BCA and BCAC, careful attention was given to ensuring complete removal of capsule and achieve a margin-free excision. The diagnosis was established through an examination of histopathological features, following the guidelines of the WHO histology classification.

## Results

Among the 1268 patients who presented with parotid gland masses and underwent parotidectomy—whether partial, superficial, or total—88 individuals received a post-surgery diagnosis, which included 81 cases of BCA and 7 cases of BCAC (Table 1).

**Table 1:** Demographics and clinical data

	Basal cell adenoma (*n* = 81)	Basal cell adenocarcinoma (*n* = 7)	p-value
Mean age (youngest-eldest)	55.1 (23–85)	68.6 (52–87)	0.056
Gender	34 male (42%) 47 female (58%)	7 female (100%)	0.076
Location	81 parotid (100%) 42 left (52%) 39 right (48%)	7 parotid (100%) 3 left (43%) 4 right (57%)	0.193
Preoperative symptoms	61 painless swelling (76.3%) 20 incidental finding (24.7%)	7 painless swelling (100%)	–
Type of parotidectomy	44 partial (54%) 35 superficial (43%) 2 total (2%)	4 partial (57%) 2 superficial (29%) 1 total (14%)	–
Radiologic size of radiologic largest dimension length (smallest-largest) (SD)	2.2 cm (0.5–17) (1.9)	2.4 cm (1.7–3.5) (0.6)	0.16
Pathologic size of tumor (smallest-largest) (SD)	2.1 cm (0.5–12.5) (1.4)	2.9 cm (2.3–3.6) (0.5)	0.011
Median time from detection to operation (shortest-longest)	35.3 months (2-241) Unknown 12 patients	28.8 months (2-121) Unknown 1 patient	0.219
Median follow up (shortest-longest)	39.1 months (1-221)*	36 months (4-121)**	0.678
Postoperative complications	3 hematoma (3.7%) 2 sialocele (2.5%) 1 partial facial nerve palsy (1.2%)	1 facial nerve palsy (14.3%)	0.081
Adjuvant treatment	None	1 chemotherapy (14.3%)	–
Relapse	None***	None****	–

SD Standard Deviation

* 40 patients (49%) had follow up period under 6 months

** 2 patients (28.6%) received radiotherapy, and 1 patient had lymph node metastasis (pT2N1M0)

*** 1 patient has received second surgery due to recurred basal cell adenoma 14 years ago at other hospital

**** 1 patient expired by cardiac arrest and 1 patient was still alive, but both had no cancer recurrence

2 patients were lost to follow up

**Fig. 2:**
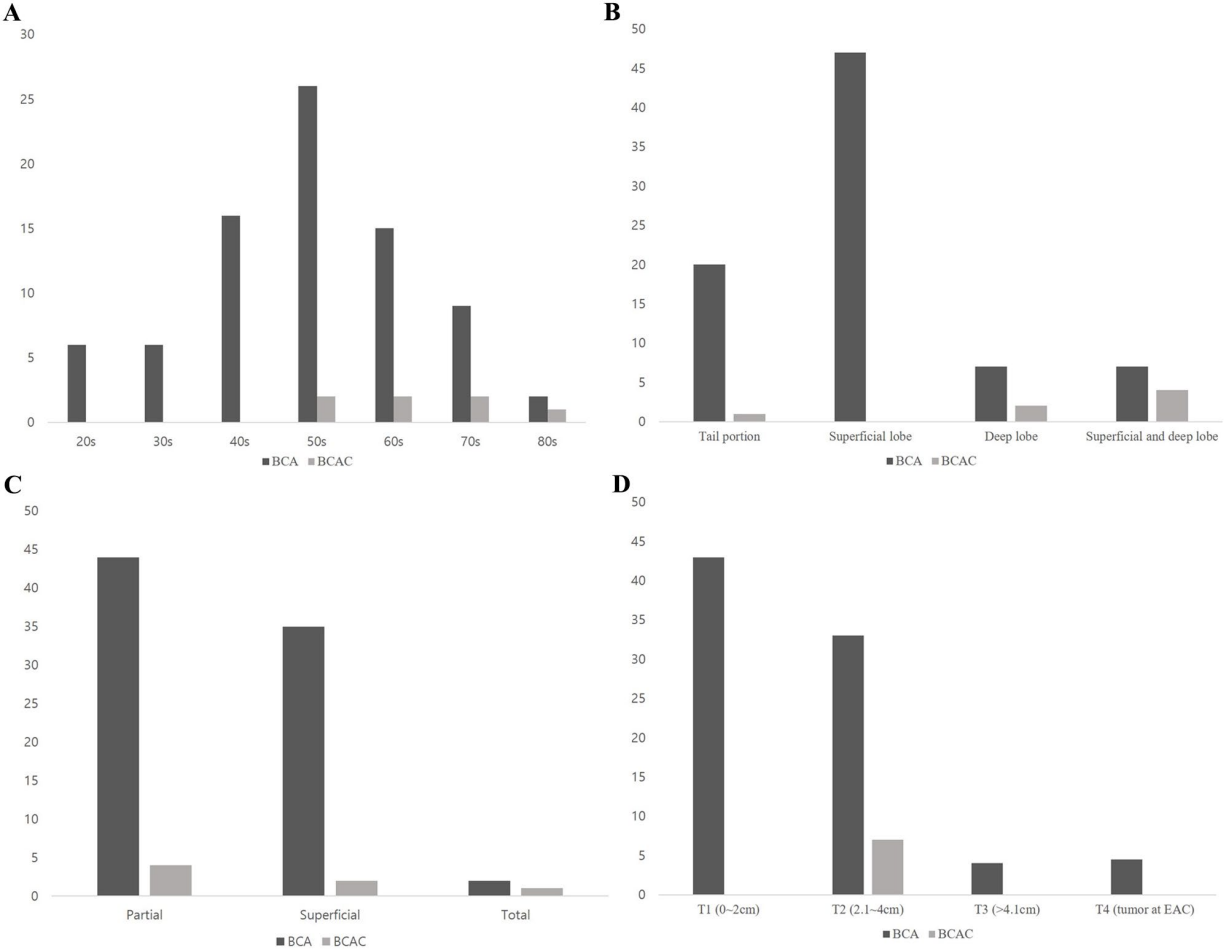
Clinical characteristics of BCA and BCAC. **A** Age distribution. **B** Tumor location. **C** Parotidectomy type. **D** T stage

A total of 81 patients were diagnosed with BCA, exhibiting a mean age of 55.1 years, ranging from 23 to 85. The age distribution was predominantly observed in the 50s, followed by the 40s and 60s, indicating a diverse age distribution ranging from the 20s to the 80s (Fig. 2A). Gender distribution included 34 males and 47 females, with a left-right distribution of 42 and 39, respectively. The mean size of BCA lesions was 1.2 cm (ranging from 0.5 to 12.5 cm). Among 81 patients, 61 presented to the hospital with painless swelling, while 20 came in as incidental findings during examinations. Tumor distribution exhibited variability, comprising 47 cases in the superficial lobe, 20 in the tail portion, 7 in the deep lobe, and 7 cases involving both the superficial and deep lobes (Fig. 2B). In terms of parotidectomy type, 44 patients underwent partial parotidectomy, 35 underwent superficial parotidectomy, and 2 underwent total parotidectomy (Fig. 2C). The mean size of lesions, measured along the longest axis using CT or MRI, was 2.2 cm, ranging from 0.5 to 17 cm. Postoperative follow-up duration averaged 31 months, with a range spanning from 1 to 221 months. Postoperative complications occurred in three cases with hematoma, two cases with sialocele, and one case with partial facial nerve palsy.

In cases of BCAC, a total of 7 individuals were diagnosed, with a mean age of 68.6, all of whom were female. The age distribution of individuals diagnosed with BCAC revealed that all subjects were aged 50 and above, exhibiting a nearly uniform distribution extending into the 80s (Fig. 2A). The left-right distribution revealed 3 cases on the left and 4 on the right. The mean size of the mass was 2.9 cm, ranging from 2.3 to 3.6 cm, and the mean size measured by radiographic imaging was 1.0 to 3.5 cm (Fig. 2D). The location of tumor consisted of both the superficial and deep lobes in four cases, with no instances of exclusive presence in the superficial lobe (Fig. 2B). Two individuals had tumors in the deep lobe, and one in the tail portion (Fig. 2B). In all cases, FNA was performed, but malignancy-indicative findings were observed only in three cases, leading to subsequent superficial or total parotidectomy. Regarding parotidectomy type for BCAC, the most common procedure was partial parotidectomy in four cases, followed by superficial parotidectomy in two cases, and total parotidectomy in one case (Fig. 2C). The median follow-up duration was 36 months, ranging from 4 to 121 months. Based on the pathological examination results of the tumors diagnosed as BCAC, the T stage for all 7 cases was pT2, indicating that they exceeded 2 cm but were less than 4 cm. Three patients with confirmed malignancy on preoperative FNA underwent neck dissection, with only one showing a positive lymph node. Chemotherapy consisting of 6 cycles using paclitaxel and carboplatin was administered to the patient with a positive lymph node. All other cases showed no metastasis to the lymph nodes. There were also no cases of BCAC where the tumor metastasized to other organs. Facial nerve palsy occurred in one case that underwent total parotidectomy. In both BCA and BCAC, preoperative facial nerve examinations showed normal findings in all cases, and no cases of recurrence or mortality were observed in follow-up period. Only one patient at the BCAC group received adjuvant treatment through chemotherapy. The above results were summarized in Table 1.

## Discussion

Classified by the WHO, benign salivary gland tumors comprise 11 different types, among which BCA is characterized as a benign unspecified entity, exhibiting an overall incidence rate ranging from 1.1 to 3.7% [4, 19]. BCAC, a rare entity comprising only 2.9% of all salivary gland carcinomas, has resulted in limited research on this specific subtype [7]. There has been a recent surge in studies focused on BCA and BCAC conducted at a single center. Rito et al. reported a single-institution-based study, presenting 41 cases of BCA and 27 cases of BCAC [18]. Tereda et al. reported on the clinical features of nine patients with BCAC, comparing them to 45 patients with BCA treated during the same period [7]. Tables 2 and 3 summarize studies that report the demographic and clinical features of BCA and BCAC, respectively. Our study, spanning over two decades, primarily focused on the demographics, tumor characteristics, and clinical outcomes of patients diagnosed with BCA and its malignant counterpart, BCAC. This study reports the largest number of BCA cases among studies conducted at a single institution, aiming to provide insights into the clinical characteristics and surgical outcomes of both BCA and BCAC.

**Table 2:** Case series of BCA previously published between 2007 and August 2022

Authors (country)	N	Mean age (range)	Gender	Mean of tumor size (cm)	Site	Histopathological subtypes	Recurrence
Wilson et al. (USA) [11]	41	58 (14–86)	F = 24 M = 17	1.9	Parotid gland = 33 Oral cavity = 6 Submandibular gland = 1 Pharynx = 1	Trabecular = 25 Membranous = 6 Solid = 5 Tubular = 5	2/30 (6.7%)
Lu et al. (China) [19]	29*	57.0 (32–83)	F = 15 M = 13	2.08	Parotid gland = 29	Solid = 17 Tubular = 6 Trabecular = 4 Membranous = 2	0
Shi et al. (China) [32]	22	51.5 (32–73)	F = 16 M = 6	1.89	Parotid gland = 22	NI	NI
Lee et al. (Taiwan) [33]	41	54** (34–81)	F = 27 M = 14	1.5**	Parotid gland = 40 Pharynx = 1	Tubulotrabecular = 23 Trabecular = 11 Solid = 4 Cribriform = 3	NI
Cordeiro et al. (Brazil) [34]	30	66.1 (42–86)	F = 21 M = 9	3	Parotid gland = 28 Submandibular gland = 1 Upper lip = 1	Trabecular = 15 Solid = 8 Tubular = 7	0***
Terada et al. (Japan) [7]	45	58 (22–80)	F = 38 M = 7	2.2	Parotid gland = 45	NI	NI
Rito et al. (Portugal) [18]	38	62.4 (NI)	F = 21 M = 17	2.1	Parotid gland = 37 Minor gland = 1	Mixed = 30 Trabecular = 8	0

BCA Basal cell adenoma, N Number of cases, F Female, M Male, NI not informed

* One case was bilateral

** Median informed

*** Patient did not present new tumor recurrence

**Table 3:** Case series of BCAC previously published between 1990 and April 2021

Authors (country)	N	Mean age (range)	Gender	Mean of tumor size (cm)	Site	Histopathological subtypes	Recurrence
Wilson et al. (USA) [11]	29	67 (40–90)	F = 17 M = 12	2.9	Parotid gland = 22 Oral cavity = 3 Pharynx = 2 Submandibular gland = 1 Sublingual gland = 1	Solid = 15 Trabecular = 8 Membranous = 4 Tubular = 2	3/18 (16.7%)
Muller et al. (USA) [16]	7	56 (46–74)	F = 6 M = 1	2.7	Parotid gland = 6 Submandibular gland = 1	Solid = 5 Membranous = 1 Solid = Membranous = 1	2 (28.6%)
Terada et al. (Japan) [7]	9	52 (27–62)	F = 6 M = 3	2.5	Parotid gland = 9	Tubular > Trabecular = 4 Trabecular = 2 Tubular = 1 Trabecular > Tubular = 1 Trabecular = Tubular = 1	0
Gutierrez et al. (USA) [21]	14*	59 (31–83)	F = 6 M = 8	NI	Parotid gland = 14	NI	0
Rito et al. (Portugal) [18]	22	62.7 (42–83)	F = 8 M = 14	3.7	Parotid gland = 10 Nasal cavity = 5 Other major = 4 Other minor = 3	NI	3/21 (14.3%)
Ellis et al. (USA) [31]	29	58 (27–92)	F = 12 M = 16**	2.1	Parotid gland = 24 Submandibular gland = 3 Unknown = 2	Solid = 19 Membranous = 6 Trabecular = 3 Tubular = 1	7 (24.1%)
Warrick et al. (Canada) [22]	4	62 (46–72)	F = 1 M = 3	3.4***	Parotid gland = 3 Nasal cavity = 1	Solid = 2 Tubular = 1****	1 (25%)

BCAC Basal cell adenocarcinoma, N Number of cases, F Female, M Male, NI not informed

* One case was bilateral

** Gender of one patient was unknown

*** Size of one case not informed

**** Histology of one case not informed

### Demographics and clinical characteristics

BCA and BCAC are both reported to have a near equal proportion of gender balance and occur over a wide age range, most commonly in the sixth to seventh decade [10, 18, 20]. In this study, what notably differs from previous reports is the diverse age distribution of BCA, ranging from the 20s to the 80s, with a peak in the 50s. For BCAC, all seven cases involved female patients. BCAC is known to originate in the parotid glands in 88% of cases, with no gender preference [8]. This suggests a potential difference in demographics concerning occurrences in Asians, indicating the need for further research in this direction. Subsequent studies are warranted for a more comprehensive understanding of these demographic variations.

Limited research has been conducted to date on the specific locations of occurrence within the parotid gland for BCA and BCAC. In our study, 47 out of 81 cases of BCA were located exclusively in the superficial lobe, whereas no BCAC cases were confined solely to the superficial lobe; instead, all cases involved the deep lobes. In terms of tumor size, 76 out of 81 BCA cases and all 7 BCAC cases were confirmed to be at T2 stage or below, indicating sizes of 4 cm or smaller. In contrast to previous reports highlighting larger sizes for BCAC, our study showed relatively smaller sizes, suggesting a potential association with the observed favorable prognosis [10, 16]. In recent times, many patients tend to seek medical attention relatively early, before the lesion reaches a considerable size. In cases where cancer is not significantly advanced, clinical differentiation becomes challenging, as there is often no clinical evidence of facial nerve involvement or infiltration into surrounding tissues [21, 22]. Our study showed that all patients presented with incidental findings or painless swelling, underscoring the importance of clinical differential diagnosis in the early stages of tumor.

### Differential diagnosis

Fine needle aspiration (FNA) has emerged as a pivotal diagnostic tool in evaluating parotid masses, providing preliminary insights before surgical intervention [9]. Studies have reported the diagnostic utility of FNA in BCA or BCAC as well [23–25]. In a recent study discussing the imaging features of 43 cases of parotid BCA, it was noted that BCAs with widespread cribriform structures and of the membranous type may undergo malignant transformation, requiring careful consideration in clinical management [17]. The study underscored the significance of FNA in distinguishing between benign and malignant conditions, identifying malignancy or suspicion of malignancy in 6 out of 9 BCAC patients. However, even in all studies highlighting the usefulness of FNA, there is an emphasis on the potential diagnostic pitfalls and the need to consistently consider the possibility of misdiagnoses. The low prevalence of BCA and BCAC contributes to these errors. In addition, BCA and BCAC have common cellular characteristics, including trabecular, tubular, solid, and membranous patterns, with frequent occurrences of mixed patterns [10]. In our study, reflecting on the cases of BCAC, four out of seven were initially approached without suspicion of malignancy, and surgery proceeded presuming BCA. This suggests that surgeons should be mindful of the potential for malignancy during the surgical procedure, even when the initial diagnosis is BCA through FNA. Therefore, it is essential to explicitly explain this possibility to the patient before surgery.

The malignant transformation of benign tumors of the salivary gland is predominantly seen in the pleomorphic adenoma, with a range of 4.5–8.5% of cases [26]. However, the occurrence of carcinomas arising in BCA is rare, with few reported cases presenting various malignant components such as adenocarcinoma not otherwise specified, salivary duct carcinoma, and basaloid carcinoma [26, 27]. In Muller et al.‘s study, a literature review of previous 65 cases of BCAC revealed that 77% developed de novo, while 23% originated from preexisting BCAs [16]. The occurrence of malignant transformations of BCA in the parotid gland is a rarely documented event [28]. In cases where such transformations do occur, their manifestation is predominantly observed as malignant basaloid tumors.

### Surgical treatments and prognosis

In the case of BCA, tumor excision including the capsule was usually performed, given its typical encapsulated nature. No instances of recurrence were observed during the follow-up period when such excision, including the capsule, was undertaken. As mentioned above, BCAC may present challenges in FNA diagnosis, and surgical findings can be similar to those of encapsulated BCA. Interestingly, even in cases where malignancy was not initially suspected, and only marginal excision was performed through partial parotidectomy, our institution observed no long-term occurrences of local recurrence or metastasis. Taking into consideration findings from other studies, when parotidectomy is performed thoroughly with complete extracapsular dissection, a positive long-term outcome can be expected [29, 30]. The results of this study indicate the potential for favorable outcomes with appropriate resection, including the capsule, especially in cases with a relatively low T stage. However, many studies have reported recurrences or unfavorable outcomes in BCAC [11, 16, 31]. Since there is still limited reporting with few studies addressing the invasive features of BCAC, future research is essential to validate this assumption. It can be inferred that in cases where excision is performed suspecting BCA, including the capsule in a safe manner is necessary for a thorough resection.

This study has several limitations. In the case of BCAC, the limited number of cases remains a constraint for comprehensive characterization of clinical features. Second, the retrospective nature of the study introduces the possibility of selection bias. A substantial number of prospective studies are required to gain a thorough understanding of the prognosis and treatment of BCA and BCAC. However, considering the rarity of reported cases for both BCA and BCAC, our study is believed to contribute as a valuable resource in furthering the understanding of the clinical characteristics of these two tumors.

## Conclusion

In conclusion, this study presents the largest single-institution dataset of BCA cases, providing comprehensive insights into the demographics, tumor characteristics, and clinical outcomes of both BCA and BCAC. Although further research is warranted, this study suggests that favorable outcomes may be achieved with appropriate parotidectomy, including the capsule, especially when the tumor is at a low T stage. Additionally, surgeons should communicate the potential for malignancy to patients, even in cases with a preoperative FNA diagnosis of BCA, and be attentive to this aspect during the actual surgical procedure.

## Data Availability

No datasets were generated or analysed during the current study.
